# Characterization of Microwave-Controlled Polyacrylamide Graft Copolymer of Tamarind Seed Polysaccharide

**DOI:** 10.3390/polym14051037

**Published:** 2022-03-04

**Authors:** Sheetal Jha, Rishabha Malviya, Shivkanya Fuloria, Sonali Sundram, Vetriselvan Subramaniyan, Mahendran Sekar, Pradeep Kumar Sharma, Srikumar Chakravarthi, Yuan Seng Wu, Neelesh Mishra, Dhanalekshmi Unnikrishnan Meenakshi, Vijay Bhalla, Sinouvassane Djearamane, Neeraj Kumar Fuloria

**Affiliations:** 1 Department of Pharmacy, SMAS, Galgotias University, Greater Noida 201310, India; sheetu.jha92@gmail.com (S.J.); rishabha.malviya@galgotiasuniversity.edu.in (R.M.); sonaliaim13@gmail.com (S.S.); neelesh.mishra38@gmail.com (N.M.); 2 Faculty of Pharmacy & Centre of Excellence for Biomaterials Engineering, AIMST University, Bedong 08100, Malaysia; 3 Faculty of Medicine, Bioscience and Nursing, MAHSA University, Jalan SP 2, Bandar Saujana Putra, Jenjarom 42610, Malaysia; drvetriselvan@mahsa.edu.my (V.S.); srikumar@mahsa.edu.my (S.C.); 4 Department of Pharmaceutical Chemistry, Faculty of Pharmacy and Health Sciences, Royal College of Medicine Perak, Universiti Kuala Lumpur, Ipoh 30450, Malaysia; mahendransekar@unikl.edu.my; 5 Accurate College of Pharmacy, Knowledge Park-III, Greater Noida 201306, India; pradeepbsr17@gmail.com; 6 Department of Biological Sciences, School of Medical and Life Sciences, Centre for Virus and Vaccine Research, Sunway University, Subang Jaya 47500, Malaysia; sengwu_21@yahoo.com; 7 College of Pharmacy, National University of Science and Technology, Muscat 130, Oman; dhanalekshmi@nu.edu.om; 8 SGT College of Pharmacy, SGT University, Budhera, Gurugram 122505, India; dean.fphs@sgtuniversity.org; 9 Department of Biomedical Science, Faculty of Science, Universiti Tunku Abdul Rahman, Kampar 31900, Malaysia; sinouvassane@utar.edu.my; 10 Center for Transdisciplinary Research, Department of Pharmacology, Saveetha Institute of Medical and Technical Sciences, Saveetha Dental College and Hospital, Saveetha University, Chennai 600077, India

**Keywords:** tamarind seed polysaccharides, grafted copolymer, acrylamide, cerric ammonium nitrate, antibacterial, thermal stability

## Abstract

The main objective of the study was to prepare tamarind seed polysaccharide grafted copolymers of polyacrylamide (TSP-g-Am) using a 3^2^ factorial design. Tamarind seed polysaccharide (TSP) was extracted, and grafted copolymer of TSP was prepared using polyacrylamide as copolymer and ceric ammonium nitrate as initiator. Various batches (F1-F9) of TSP-g-Am were prepared, among which F1 showed highest grafting efficiency; hence, the prepared TSP-g-Am (F1) was evaluated for grafting efficiency, conversion, effect of initiator and further characterized using SEM analysis, contact angle determination, DSC analysis, swelling index, swelling and deswelling, and chemical resistance. The contact angle of TSP was found to be 81 ± 2, and that of TSP-g-Am (F1) was found to be 74 ± 2, which indicates that the wetting ability of the grafted copolymer was less than that of the native polymer. The results of thermal analysis indicated that TSP-g-Am had a more stable molecular structure than TSP. The morphology of the grafted polymer was observed from SEM images, and it was observed that the particles was asymmetrical. Antimicrobial activity was also found in the grafted copolymer. The present study concludes that the TSP-g-Am showed an excellent performance in thermal stability and swelling capacity compared with TSP. The detailed structural characteristics, as well as the excellent thermal stability and swelling capacities, will make it beneficial to use the synthesised copolymer as a precursor for the production of large-scale eco-friendly advanced materials with a wide range of applications, acting as a stabiliser, thickener, binder, release retardant, modifier, suspending agent, viscosity enhancer, emulsifying agent, or carrier for novel drug delivery systems in oral, buccal, colon, and ocular systems, and in nanofabrication and wound dressing, and it is also becoming an important part of food, cosmetics, confectionery, and bakery.

## 1. Introduction

Natural polymers have been a saviour in the pharmaceutical industry during the last few decades. As reported in various studies, they are economical, as well as exhibiting many benefits, such as biodegradability, ease of availability, non-toxicity, etc. [1,2]. Despite having such attractive characteristics, natural polymers possess drawbacks such as uncontrolled hydration, microbial contamination, and batch-to-batch variation. To overcome these limitations and to impart new and improved properties, it is necessary to chemically or physically modify such polymers [3,4]. As reported in a study by Thakur et al., the chemical modification of gums not only minimizes these drawbacks, it also enables their use for specific drug delivery purposes [5]. The modification of natural polymers enhances their drug delivery properties and versatility. The study by Bhosle et al., showed that the modification or grafting of polymers has been attracting increasing attention for the production of tuneable polymeric materials [6]. There are various methods for the modification of gums, like grafting, cross linking, derivative formation, and polymer–polymer binding.

As per Mittal et al., and Kumar et al., among the various modification techniques, grafting is one of the most promising methods, and involves microwave irradiation [7,8]. The grafting of polyacrylamide has been used for the modification of natural polysaccharides. Modified polysaccharides formed in this way are known as graft copolymers. Graft copolymers are useful due to their remarkable properties. Graft copolymers consist of a macromolecular network in which one or more of blocks are well connected as the side chains to the main backbone of a polymer, generating many interesting properties. After grafting, the host polymer attains many desirable properties from the grafted monomer linkages [9].

The modified product formed using this technique has a variety of advantages. For example, acrylamide and methacrylic grafted gum ghatti were found to possess cation exchange properties, act as super absorbents, and possess better gelling properties than the native form [10], while also exhibiting improved thermal stability [11]. In the study of Singh et al., a novel polymeric flocculant was developed by graft copolymerization of polyacrylamide with acrylic acid using γ irradiation technique [12]. Gum Arabic is another polysaccharide, and consists of glucuronic acid with galactose, arabinose and rhamnose. Gum Arabic has been used as an emulsifying agent in the food industry, as well as in pharmaceutical formulations at a concentration of 15%. It has also been found that the chemical modification of gum increases its emulsifying properties [13]. Different studies have shown that the encapsulation efficacy of gum can be increased by esterification using alkane- or alkene-substituted dicarboxylic acid anhydride, e.g., octenyl succinic anhydride. It has also been observed that encapsulation property depends upon length as well as the amount of alkyl chains attached to gum [14,15,16].

According to Kumar et al., graft copolymerization is a significant technique for adding advanced properties to the polymeric backbone. It is a chemical technique that imparts desirable features to natural fibres without affecting their inherent behaviours [8]. Graft copolymers also show significant pharmacological activities. Mishra et al. and Dholakia et al. reported that grafted products can significantly improve the antibacterial activity of polymers [17,18]. Maji et al. showed that polyacrylamide grafted polymer possesses sustained release action in simulated biological fluid due to the increased solubility time of the grafted polymer [19].

*Tamarindus indica* L. (Tamarind), commonly known as Imli, has been attracting increasing attention for a wide potential range of applications in the pharmaceutical and biomedical industries. Tamarind seed provides tamarind seed polysaccharide, which is a rich source of glucose, xylose and galactose, in the ratio of 3:2:1 [20,21,22]. The isolation and characterization of TSP (tamarind seed polysaccharide) is a very simple process, and is highly cost-effective with respect to its yield. Polyacrylamide grafted TSP can be used for the formulation of sustained-release dosage forms. In the present investigation, microwave irradiation-controlled modification of TSP was carried out using polyacrylamide as the copolymer and ceric ammonium nitrate as the initiator. Furthermore, the graft copolymer was characterized as pharmaceutical excipient on the basis of various technological parameters.

## 2. Materials and Methods

### 2.1. Materials

Kernels of Tamarind were purchased from the local market in Greater Noida, Uttar Pradesh, India. Acrylamide, cerric ammonium nitrate, ethanol and acetone were of analytical reagent grade (CDS, Delhi, India). All the reagents were used without prior purification. Double-distilled water was used as a solvent throughout the experiment.

### 2.2. Method

Extraction of TSP: Extraction of TSP was carried out according to procedure followed by Malviya et al. [23,24]. The required amount of tamarind seeds was dried at 40 °C for 10 min and the brown coatings were removed. Furthermore, distilled water was added to the beaker containing uncoated seeds and stirred for 2–3 h at 40 °C to prepare the slurry. The slurry was further kept under mechanical stirrer for uniform distribution. Slurry containing beaker was kept at 40 °C for 3 h with continuous stirring. Slurry was then filtered using muslin cloth and transferred into a beaker. To precipitate and filtrate the slurry, ethyl alcohol was added to double amount of slurry. The precipitate was washed again with ethyl alcohol. The washed precipitate was dried in hot air oven at 40 °C until constant weight was obtained. The size of the dried product was reduced using a domestic mixer grinder, and the powder was passed through #45 mesh. The powder was kept in an airtight container for further study. All physicochemical characterizations carried out are reported in Malviya et al. [25].

#### 2.2.1. Synthesis of Polyacrylamide Grafted Tamarind Seed Polysaccharide

##### Factorial Design

A 32-factorial design was used for the preparation of polyacrylamide grafted copolymer of tamarind seed polysaccharide (TSP-g-Am). In the present research, quantity of CAN (gm) and microwave exposure (time) were considered as independent variables; and for dependent variables, grafting efficiency (%) and conversion (%) were selected. Three levels were selected for every independent variable as shown in Table 1. The NCSS 21 software (Trial version 15/08/2021) was used for the of designing of surface response curve.

Synthesis of polyacrylamide grafted copolymer of TSP was achieved by the free radical-induced grafting methodology [26,27,28,29]. The required quantity (1 gm) of TSP was added to a beaker containing 30 mL of doubled distilled water to make a homogeneous solution. Polyacrylamide (6 gm) was added to another beaker with 25 mL of double-distilled water. After proper mixing, the polyacrylamide solution was added to the beaker containing aqueous polymer solution. The aqueous solution of 30 mL CAN (0.3g) was added to the above dispersion and stirred for another 30 min. After proper mixing, the beaker was kept overnight. The dispersion was irradiated using a microwave for 30 s at 100 W for different times, as shown in Table 1. The beaker was irradiated by alternating 1 min heating and 1 min cooling. The irradiated sample was then precipitated using acetone. The precipitated product was further washed with 20% aqueous ethanol in order to remove unreacted homopolymer. The thus-formed grafted polymer was dried in an oven at 40 °C. After drying, the size reduction of modified polymer was carried out using a domestic mixer grinder and the powder was passed through #45 mesh. The powder was kept in an airtight container for further study.

Characterization of TSP-g-Am: the synthesized polyacrylamide graft copolymer was characterized using the following parameters:

Grafting: to assess the efficiency of the grafting process, different grafting parameters, including grafting (%), grafting efficiency (%), and conversion (%), were determined using Equations (1)–(3), respectively [24,29,30,31].

(1) $$\mathrm{Grafting}\;(\%)=\frac{\left(w_{1}-w_{0}\right)\times 100}{w_{0}}$$

(2) $$\mathrm{Grafting}\;\mathrm{efficiency}\;(\%)=\frac{\left(w_{1}-w_{0}\right)\times 100}{w_{2}}$$

(3) $$\mathrm{Conversion}\;(\%)=\left(\frac{w_{1}}{w_{0}}\right)\times 100$$

where w_0_ = weight of TSP, w_1_ = weight of TSP-g-Am, w_2 =_ weight of polyacrylamide used.

Characterization of TSP and TSP-g-Am: among the various grafted batches, F1 showed the maximum grafting efficiency and conversion, and so was selected for further characterization. Both native and grafted polymers were characterized in terms of the following parameters:

Contact angle determination: contact angle determination was performed to identify the ability of wetting of the native polymer, and this was compared against the TSP-g-Am (F1). Copper plate was used for the drop formation. Then, the drop was vacuum dried and the plate was kept under the NIKON microlens (Tokyo, Japan) at a distance of 22 cm (object piece). A PHANTOM HIGH PEAK camera-1300 (Wayne, NJ, USA) was used for the whole procedure [32].

Differential scanning calorimetry: the DSC of TSP and TSP-g-Am (F1) was recorded using a Shimadzu DSC-60 (Kyoto, Japan) in the temperature range of 0–400 °C at a heating rate of 10 °C per minute in a nitrogen environment [33].

Scanning electron microscopy: the SEM of the TSP and TSP-g-Am (F1) was analysed by using Zeiss EVO 18 analyser (Jena, Germany). The sample was gold coated and mounted in the sample holder. The surface morphology of the polymers was determined at different magnifications [34].

Swelling index: for the determination of swelling index, 500 mg of the grafted polymer (F1) was placed on a butter paper (size 5 × 5 cm^2^) in a petri dish that was immersed in 25 mL of double-distilled water. After 1 h, the weight of the swelled polymer was measured on a digital balance and the swelling index was determined by using Equation (4) [35].

(4) $$\mathrm{SI}\;\left(\%\right)=\frac{\left(w_{1}-w_{0}\right)\times 100}{w_{1}}$$

where w_2_ = weight of swelled polymer, w_1_ = initial weight of polymer.

Swelling deswelling study: the required amount of graft copolymer (F1) was weighed and wrapped in muslin cloth. The whole set up was then immersed in a beaker containing 40 mL 0.1 N HCl and kept for 20 min. After 20 min, the same setup was immersed in a beaker containing 40 mL 1N NaOH. This process was continued for 120 min. The swelling index was then determined, and a graph was plotted between swelling index and time.

Chemical resistance test: to determine the chemical resistance of the graft copolymer, 500 mg of the (F1) was immersed in 25 mL of 0.1 N HCl and 1 N NaOH solution separately kept in a petri dish. The swelled polymer was then recovered and wiped off with tissue paper and dried at 40 °C until constant weight was achieved. The observations were noted at 12 h [31]. The weight loss (%) was determined by Equation (5).

(5) $$\mathrm{Weight}\;\mathrm{loss}\;\left(\%\right)=\frac{\left(w_{i}-w_{f}\right)\times 100}{w_{i}}$$

where w_f_ = weight of swelled polymer, w_i_ = initial weight of polymer.

Test for antimicrobial efficacy: the disc diffusion method was used for the evaluation of antimicrobial activity of TSP and TSP-G-Am (F1). The test microorganism was obtained from the Department of Medical Lab Technology, School of Medical and Allied Sciences, Galgotias University, Greater Noida, India, and comprised the Gram-negative bacteria *Escherichia coli* (E. coli) and the fungus *Aspergillus niger*. Culture of test organism was performed using sterilized nutritive agar medium. E. coli and *Aspergillus niger* were cultured for 24 h at 30 °C and 48 h at 30 °C, respectively. TSP and TSP-G-Am were dissolved and diluted in double-distilled water to prepare 1, 0.5, and 0.25 mg/mL solutions. Solutions were poured onto 5 mm discs and incubated for the next 24 h. Activity assays were repeated for three times. After incubation, inhibition zone was measured in millimetres and antimicrobial activity of F1 was compared with native polymer, i.e., TSP.

## 3. Results and Discussion

**Figure 1:**
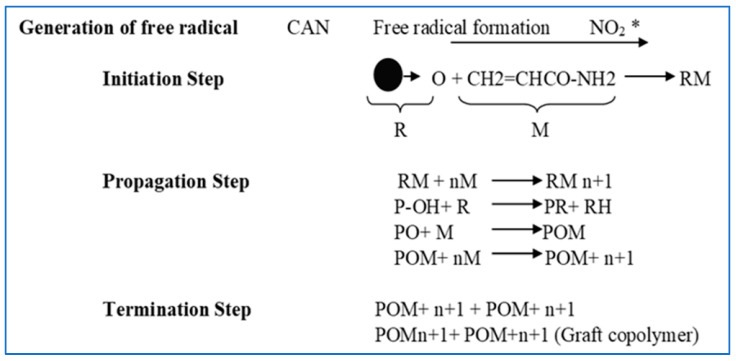
Schematic diagram of polyacrylamide grafting.

Polyacrylamide grafting over a TSP backbone was successfully caried out by using microwave irradiation. In the process of grafting, ceric ammonium nitrate was used as initiator to form free radicals. CAN forms NO_2_ free radicals that further react with the -OH group of TSP. This further leads to the formation of free radicals over the TSP backbone. NO_2_ free radicals also react with polyacrylamide, leading to the formation of free radical formation over polyacrylamide. Grafting was initiated when TSP free radicals reacted with polyacrylamide free radicals. Figure 1 depicts the grafting of polyacrylamide over the TSP backbone. The native polymer with a free oxygen group was attached with the monomer, with polyacrylamide forming a polymer–monomer complex (RM). Proceeding to the next step, propagation, the RM complex was again introduced to another monomer chain forming another complex, and so on. The thus-formed TSP graft copolymer was formed along with some homopolymer, which was removed soon in the washing step. Ikhuoria et al. also synthesized graft copolymer of acrylonitrile onto cassava starch by ceric ion-induced initiation [36].

Various characteristics of grafted tamarind seed polymer is summarised in Table 2.

### 3.1. Factorial Design

In the present research, a 32-factorial design was used to evaluate the effect of the independent variables (quantity of CAN and microwave exposure time) on the grafting efficiency (%) and conversion (%) of grafted copolymer (dependent variables). The reduced equation for measuring the response (grafting efficiency and conversion) with statistical significance for a 32-factorial design is as shown in Equation (6).

(6) $$y=b_{0}+b_{1}X_{1}+b_{2}X+b_{12}X_{1}X_{2}+b_{11}X_{12}+b_{22}X$$

where y = response of variables (dependent variables); b_0_ = arithmetic mean response of nine batches; b_1_ = estimated coefficient of factors x_1_. The coefficients for the corresponding liner effect (b_1_ and b_2_), interaction (b_12_) and quadratic effect (b_11_ and b_22_) were determined from the results of the experiment.

On the basis of the results, Equation (6) has been solved to determine the effect of the quantity of CAN and microwave exposure time on the grafting efficiency (Equation (7)) and conversion (Equation (8)) of nanoparticles.

GE (%) = 64.27+13.18(X_1_) + 10.81(X_2_) − 2.44(X_1_X_2_) + 9.03(X_1_^2^) (7)

C (%) = 8917 − 16.06(X_1_) + 17.89(X_2_) + 4.22(X_1_X_2_) + 8.94(X_1_^2^) + 5.56(X_2_^2^) (8)

**Figure 2:**
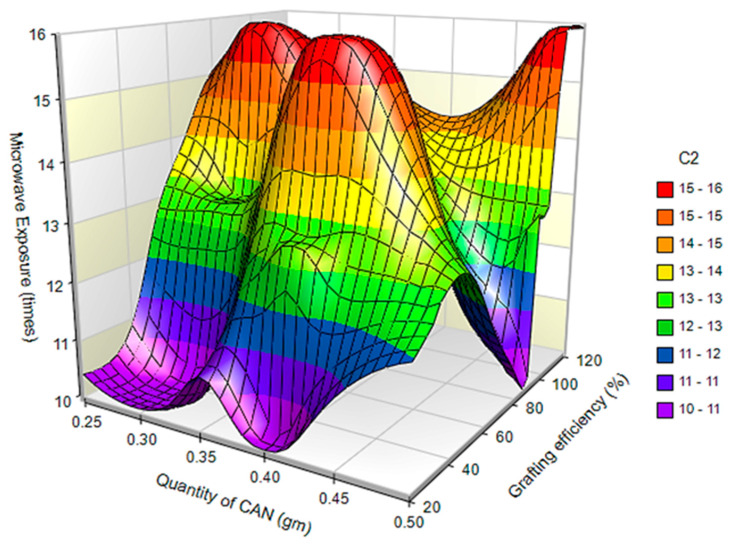
Surface plot for effect of independent variables on grafting efficiency.

**Figure 3:**
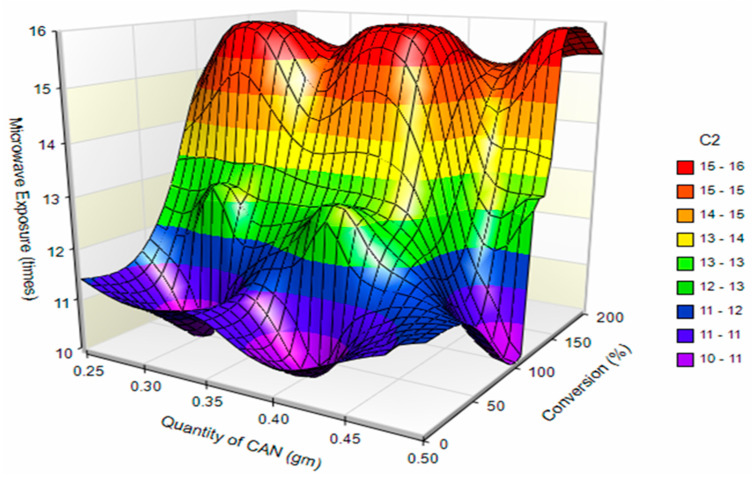
Surface plot for effect of independent variables on conversion of polymer.

Surface plots for the effect of the independent variables on grafting efficiency and conversion of polymer are presented in Figure 2 and Figure 3, respectively.

### 3.2. Effect of Initiator on Grafting

Radiation technique is a very important aspect of polymer modification; in addition, all grafting techniques require an initiator [37]. Table 1 and Table 2 show the effect of various initiator concentrations on grafting. It can be observed from the obtained data that the maximum percentage of grafting occurred at a concentration of 0.5 gm, i.e., 667.8%. In the study of Lele et al., the authors showed that the nature of the initiator has an effect on grafting [38]. Once a certain initiator concentration has been achieved, an increase in initiator level will not increase the conversion of grafted monomer. These results on the effect of the initiator on grafting were also supported by Pati et al., who showed that a further increase in initiator concentration leads to a decrease in the grafting percentage of the monomer. They reported that ceric ion at higher concentrations causes the termination of grafted polymeric chain growth, since ceric ion is a very good terminator [39].

### 3.3. Effect of Time of Exposure on Grafting

Grafting efficiency and grafting percentage were evaluated as time variables, and the results are shown in Table 2. The grafting reaches its optimum level when both of these parameters have been optimized; similarly, the grafting is worse when these two parameters are at their lowest values. Pati et al. also reported in their study that the percentage of grafting was found to increase linearly with time, and then to remain approximately constant [39].

### 3.4. Contact Angle Determination

The contact angle was determined to identify the wetting ability of the native polymer. Copper plate was used for the drop formation. Then, the drop was vacuum dried and the plate was placed under a NIKON microlens at a distance of 22 cm (object piece). A PHANTOM HIGH PEAK camera-1300 was used for the whole procedure. The contact angle of the native TSP was found to be 81 ± 2, and that of TSP-g-Am (F1) was found to be 74 ± 2. As discussed in the study by Malviya et al., contact angles less than 90° indicate favourable wetting and good spreading of liquid over the surface; however, contact angles greater than 90° indicate unfavourable surface wetting and the formation of the liquid into compact droplets on a surface. The solubility of the polymer is inversely proportional to the contact angle. The contact angles of the native polymer and the grafted polymer were found to be 75 ± 3 and 81 ± 4, respectively, in the study by Malviya et al. [40]. With increasing contact angle, the wetting ability of the polymer was found to decrease. Therefore, in this case, the wetting ability of the grafted copolymer was less than that of the native polymer.

### 3.5. DSC Analysis of Polymers

**Figure 4:**
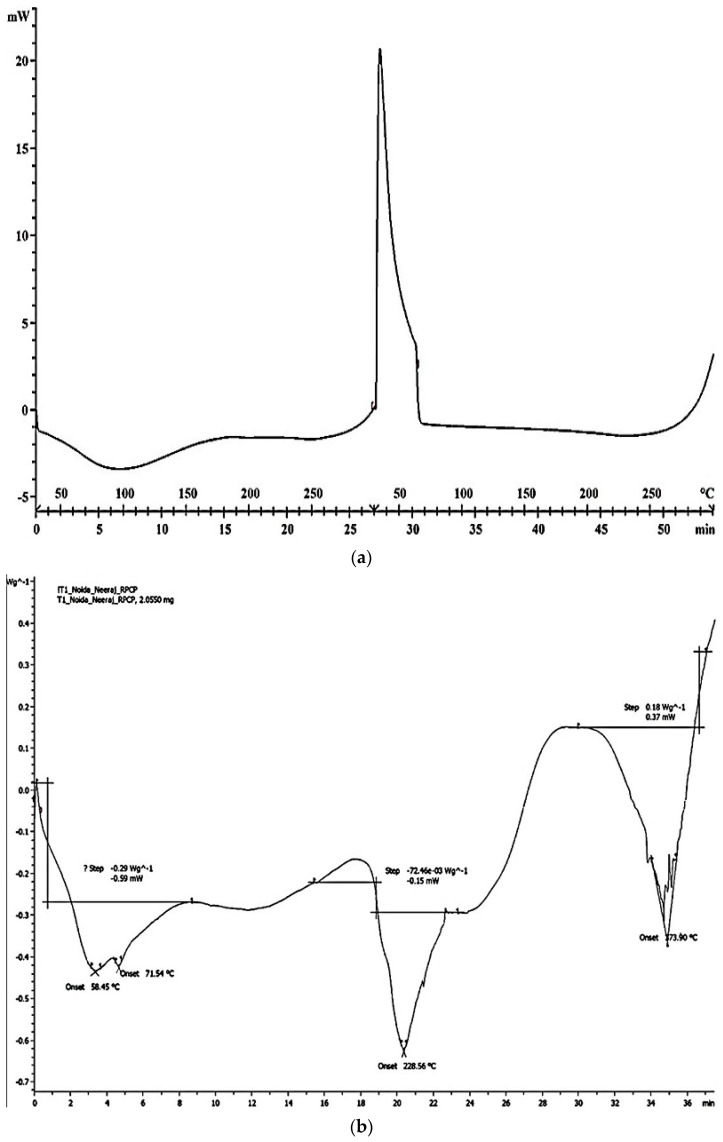
DSC of (**a**) TSP and (**b**) TSP-g-Am (F1).

The peaks showing the melting point of the native TSP and TSP-g-Am (F1) are shown in Figure 4a,b. In the case of TSP, a sharp exothermic peak evolves at 350 °C, demonstrating its crystalline nature. Meanwhile, in the case of grafted copolymer, the polymer can initially be observed to melt sharply due to the loss of absorbed moisture at the beginning of the heating, followed by three different endothermic peaks as well as two exothermic peaks. The first lower peak at 373 °C, representing the glass transition temperature, indicates that it is brittle and hard, as the peak is below the glass transition temperature. The second peak possesses an endothermic nature as it is below the transition temperature. In the study of Shi et al., two exothermic peaks were observed at 250 and 487 °C in native polysaccharides, which may be attributed to the decomposition of the branches (250 °C) and the main chain (487 °C) of the polysaccharide. However, grafted ones exhibit no obvious exothermic peak throughout the whole decomposition process [41]. Based on the above observation, a higher thermal stability and lower exothermic properties indicate that the molecular structure of the grafted copolymer is more stable, mainly due to the grafting of acrylamide onto the polysaccharide molecules of TSP.

### 3.6. Surface Morphology

**Figure 5:**
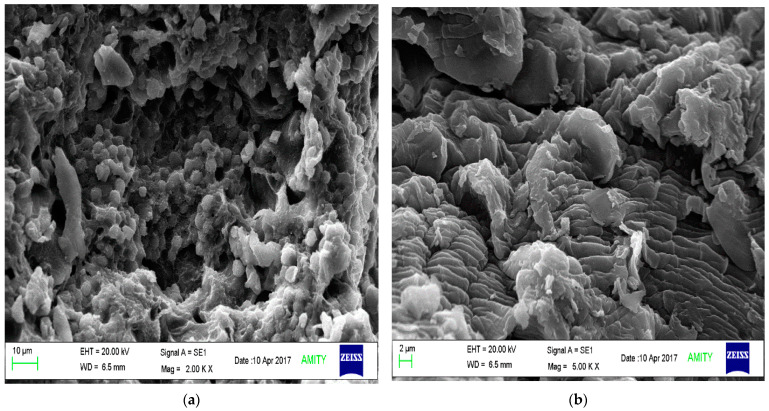
Scanning electron microscopy of (**a**) native TSP and (**b**) grafted copolymer (F1).

SEM analysis was performed for the determination of the structure morphology of the native and modified samples. As discussed by author in previous publication, the SEM of native polymer shows presence of two types of particles: smaller-sized particles with rough rounded edges and larger-sized particles of irregular shape with a smooth surface [25]. The SEM images of the grafted polymers is shown in Figure 5. The SEM images of the grafted copolymer (F1) reveal that some particles were asymmetrical in shape, while others possessed a layered surface. Similar results were observed in the study by Sen et al. using SEM, thus supporting this characteristic of grafted TSP [42].

### 3.7. Swelling Index

Swelling index is defined as the amount of solvent, either aqueous or non-aqueous, that can be absorbed into or entrapped within the porous surface. Grafting increases the porosity of the polymer, and so the water is entrapped within it, making it swell. The results are shown in Table 2. As also reported in various studies, the porous structure of grafted copolymer TSP-g-Am may also play an important role in the water absorption process, greatly enhancing the swelling capacity [41]. Due to the cross-linked behaviour of the polymers, the solvent becomes entrapped, leading to the swelling of the polymers.

### 3.8. Swelling and Deswelling Study

**Figure 6:**
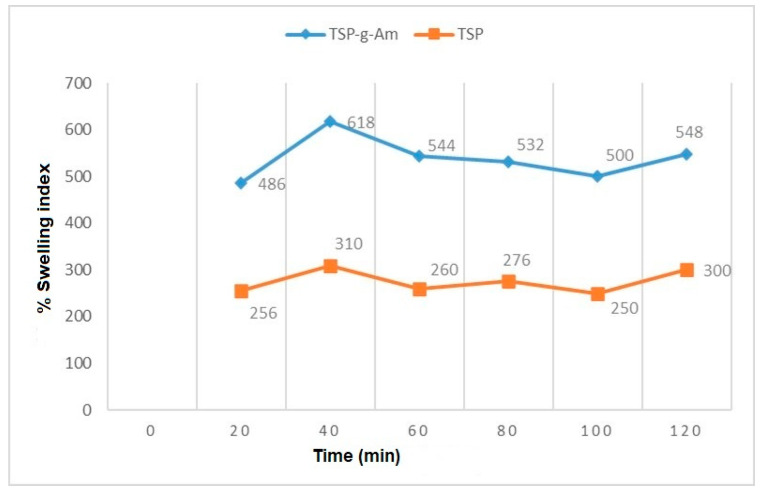
Plot showing swelling and deswelling studies on the grafted polymer.

The main mechanism involved in swelling and deswelling depends on two pH values. The remarkable swelling changes are due to the presence of different interacting species, which are dependent on the pH of the swelling medium. Under acidic conditions, the swelling is controlled mainly by the amino group of the polyacrylamide chain. It gets protonated, and the increased charge density on the polymer enhances the osmotic pressure inside the gel particles. However, under very acidic conditions, a charge screening effect of the gegenion occurs, shielding the charge from the ammonium cations and preventing efficient repulsion. As a result, a remarkable decrease in equilibrium swelling is observed. In basic medium, the amide group of polysaccharide is hydrolysed and converted into carboxylate ion (COO–), resulting in COO—COO– ion repulsion, which causes increased swelling [43]. Hence, in the presence of hydrochloric acid, the H+ ions in the HCl attach to the NH2 groups, converting them into NH4+ groups, which in turn makes the compound bigger in terms of size. This leads to the chain opening, making more space for the penetration of the water content, therefore reducing its weight. Meanwhile, in the case of NaOH, the OH group does not liberate any ions to make space. Rather, it is entrapped in the same chains, and swells in the given time duration. This increases the weight of the polymer in 1 N NaOH solution compared to 0.1 N HCl solutions. Figure 6 shows the swelling and deswelling studies on the grafted polymer.

### 3.9. Chemical Resistance

It was observed that the batch F1 showed the lowest weight loss (%) in the presence of 0.1 N HCl and 1 N NaOH, as shown in Table 2. It was concluded that the batch showing the highest grafting (%) was resistant to acidic and basic environment, as grafting inhibits chemical attack on the copolymer. It can also be concluded that the total life span of the polymer increases due to grafting. Cross linking of the polymer limits the penetration of external chemicals into the polymeric backbone, hence preventing degradation.

### 3.10. Antimicrobial Effect

The antimicrobial activity of native TSP (N1) and TSP-g-Am (F1) was determined against *Escherichia coli* (*E. coli*) and *Aspergillus niger* (*A. niger*), and clear zones of inhibition were observed. Based on the antimicrobial data given in Table 3, it can be concluded that grafted copolymer has a better antimicrobial effect than the native polysaccharides. The results of the present study are also supported by the research work of Malviya et al., who reported improved resistance of grafting over a polysaccharide backbone against microbes [40].

## 4. Conclusions

Tamarind gum graft copolymers of polyacrylamide were successfully synthesized by using ceric ammonium nitrate as initiator and under the control of microwave exposure. UV-visible spectral analysis, contact angle measurement, and SEM studies easily proved the grafting process. TSP-g-Am showed relatively higher swelling index values than TSP. The thermal analysis indicated the different stages of degradation of the grafted copolymer and also confirmed that TSP-g-Am had a stable molecular structure compared to TSP. The research also showed that the grafted copolymer had antibacterial activities. The present study establishes that polyacrylamide grafted copolymer of TSP showed better results than the native copolymer. Hence, on the basis of all of the above observations, it can be concluded that the polyacrylamide grafted copolymer of TSP has better application potential, and could be used as a pharmaceutical excipient. The present study recommends that the grafted polymers be further evaluated for toxicity using suitable animal models.

## Figures and Tables

**Table 1:** Details of independent variables for the synthesis of graft copolymers.

S. No.	Batch	Quantity of CAN (gm)	Microwave Exposure
1	F1	0.5	16
2	F2	0.3	16
3	F3	0.4	16
4	F4	0.5	13
5	F5	0.3	13
6	F6	0.4	13
7	F7	0.5	10
8	F8	0.3	10
9	F9	0.4	10

**Table 2:** Various evaluation data of grafted tamarind seed polymer [37].

Batch	Grafting (%)	Grafting Efficiency	Conversion (%)	Swelling Index (%)	Chemical Resistance (0.1N HCl)	Chemical Resistance (1N NaOH)
F1	667.8	111.2	152	97	3.9	2.5
F2	464	77.4	104	73.10	4.2	3.9
F3	587	53.4	124	89	5.7	5.1
F4	558	93.9	119	58	7.7	3.8
F5	385.5	64.2	63.5	87	6.5	5.2
F6	209	34.9	53	92	6.4	4.1
F7	531	88.5	102	90.6	5.7	2.6
F8	196	32.7	50	88.4	3.8	7.6
F9	140	23.4	35	91.1	4.2	5.5

**Table 3:** Antimicrobial activity of native TSP (N1) and TSP-g-Am (F1).

Formulation	Concentration in mg/mL	Zone of Inhibition in mm	
		*E. coli*	*A. niger*
N1	0.25	0.116 ± 0.001	0.109 ± 0.003
	0.5	0.120 ± 0.003	0.121 ± 0.003
	1	0.126 ± 0.003	0.128 ± 0.002
F1	0.25	0.289 ± 0.002	0.218 ± 0.002
	0.5	0.326 ± 0.002	0.318 ± 0.002
	1	0.427 ± 0.001	0.420 ± 0.003

## Data Availability

The data presented in this study are available in the article.
